# Supplementary material for: CAVPENET Peptide Inhibits Prostate Cancer Cells Proliferation and Migration through PP1γ-Dependent Inhibition of AKT Signaling
Source: Pharmaceutics. 2024 Sep 12;16(9):1199. doi: 10.3390/pharmaceutics16091199 (PMC11434739; doi:10.3390/pharmaceutics16091199)
Supplement: Supplementary file 1 [file pharmaceutics-16-01199-s001.zip › Supplementary Table S1.pdf]

Supplementary Table S1. Percentage of contacts of **(A)** CAVPENET control and **(B)** CAVPENET to PP1. A intermolecular contact was considered when the residues were in a distance less than 4 Å.

A

		**CAVPENET control**							
		**V1**	**V2**	**D3**	**F4**	**I5**	**K6**	**E7**	**D8**
**PP1 sequence**	**K60**	0,00	0,00	0,00	0,00	0,00	0,00	0,00	0,00
	**Y78**	0,00	0,00	0,00	0,00	0,00	0,00	4,13	2,31
	**D166**	0,02	0,00	0,00	0,00	0,00	0,00	0,00	0,00
	**K168**	53,08	79,42	0,03	0,00	0,00	0,00	0,00	0,00
	**I169**	0,05	99,48	0,00	0,00	0,00	0,00	0,00	0,00
	**L236**	3,03	0,00	0,00	0,00	0,00	0,00	0,00	0,00
	**L241**	0,10	0,00	0,00	0,00	0,00	0,00	0,00	0,00
	**D242**	66,96	99,98	0,00	0,46	0,00	0,00	0,00	0,00
	**L243**	2,53	99,95	0,01	97,35	0,00	0,00	0,00	0,00
	**C245**	0,00	0,00	0,00	0,03	0,00	0,00	0,00	0,00
	**D253**	0,00	0,00	0,00	0,00	0,00	0,23	0,38	0,01
	**Y255**	0,00	0,00	0,00	0,94	1,30	9,41	19,36	5,45
	**F257**	0,00	0,26	0,00	98,91	0,89	1,19	2,04	0,07
	**K260**	5,36	0,00	0,00	0,00	0,00	0,00	0,11	0,01
	**R261**	6,20	1,61	13,45	94,02	6,83	16,90	15,03	0,32
	**Q262**	9,20	1,27	0,01	4,07	0,00	0,00	0,00	0,00
	**L263**	0,00	0,00	0,00	0,02	0,00	0,00	0,00	0,00
	**V264**	0,00	0,00	0,00	58,63	0,00	0,00	0,00	0,00
	**M283**	0,00	1,17	0,00	72,83	0,00	0,00	0,00	0,00
	**S284**	0,00	0,00	0,00	0,00	0,02	0,00	0,01	0,01
	**V285**	0,00	2,07	0,00	0,00	0,00	0,00	0,00	0,00
	**D286**	0,00	0,00	0,00	0,00	8,03	0,00	0,00	0,00
	**T288**	65,63	1,71	0,52	0,00	0,00	0,00	0,00	0,00
	**L289**	82,59	99,58	94,88	0,00	0,00	0,00	0,00	0,00
	**M290**	30,87	34,34	99,92	56,65	96,53	23,11	0,00	0,01
	**C291**	0,00	99,39	99,95	99,99	97,98	0,00	0,00	0,00
	**S292**	0,00	0,00	0,00	30,02	96,22	6,64	6,81	1,00

	**F293**	0,00	0,00	0,00	98,70	40,47	9,44	15,85	6,10
	**Q294**	0,00	0,00	0,00	0,00	0,45	0,16	7,96	3,40
	**I295**	0,00	0,00	0,00	0,00	0,06	5,87	13,65	8,50
	**K297**	0,00	0,00	0,00	0,00	0,00	0,03	1,66	1,83

B

		**CAVPENET**							
		**V1**	**V2**	**K3**	**I4**	**D5**	**F6**	**E7**	**D8**
**PP1 sequence**	**Y78**	0,00	0,00	0,00	0,00	0,00	0,06	11,41	1,14
	**K168**	46,76	77,02	0,05	0,00	0,00	0,00	0,00	0,00
	**I169**	0,00	97,43	0,00	0,07	0,00	0,00	0,00	0,00
	**L236**	4,04	0,00	0,00	0,00	0,00	0,00	0,00	0,00
	**H237**	0,09	0,00	0,00	0,00	0,00	0,00	0,00	0,00
	**L241**	0,64	0,00	0,00	0,00	0,00	0,00	0,00	0,00
	**D242**	67,45	99,96	0,32	1,73	0,00	0,00	0,00	0,00
	**L243**	1,42	99,82	5,35	88,37	0,00	0,00	0,00	0,00
	**C245**	0,00	0,00	0,00	0,01	0,00	0,00	0,00	0,00
	**D253**	0,00	0,00	0,00	0,00	0,00	0,00	0,09	0,14
	**Y255**	0,00	0,00	0,00	0,79	0,66	57,07	15,43	9,65
	**F257**	0,01	2,68	0,00	99,43	8,94	54,84	4,25	2,91
	**K260**	7,27	0,17	0,00	0,00	0,00	0,01	0,06	0,59
	**R261**	7,83	7,86	1,77	92,31	19,51	67,34	24,72	15,49
	**Q262**	21,49	11,02	4,56	8,34	0,00	0,27	0,00	0,00
	**V264**	0,00	0,00	0,00	24,79	0,00	0,00	0,00	0,00
	**M283**	0,00	3,90	0,00	27,72	0,00	0,00	0,00	0,00
	**S284**	0,00	0,00	0,00	0,00	0,00	0,00	0,15	0,00
	**V285**	0,00	1,43	0,00	0,00	0,00	0,00	0,00	0,00

	**D286**	0,00	0,00	0,00	0,00	0,28	0,18	0,03	0,02
	**T288**	46,25	2,67	8,07	0,00	0,00	0,00	0,11	0,00
	**L289**	63,44	95,96	83,75	0,01	0,00	0,00	0,00	0,00
	**M290**	12,81	21,45	94,59	35,88	77,07	10,58	1,93	0,08
	**C291**	0,00	98,88	99,81	99,99	98,87	1,93	0,00	0,00
	**S292**	0,00	0,00	0,00	34,30	97,51	24,17	21,99	0,14
	**F293**	0,00	0,13	0,03	98,18	71,43	56,61	29,22	0,46
	**Q294**	0,00	0,00	0,00	0,00	0,11	2,19	26,83	4,97
	**I295**	0,00	0,00	0,00	0,00	0,00	25,78	27,63	17,64
	**K297**	0,00	0,00	0,00	0,00	0,00	0,08	7,66	10,47
