# Supplementary material for: CAVPENET Peptide Inhibits Prostate Cancer Cells Proliferation and Migration through PP1γ-Dependent Inhibition of AKT Signaling
Source: Pharmaceutics. 2024 Sep 12;16(9):1199. doi: 10.3390/pharmaceutics16091199 (PMC11434739; doi:10.3390/pharmaceutics16091199)
Supplement: Supplementary file 1 [file pharmaceutics-16-01199-s001.zip › Supplementary figures.pdf]

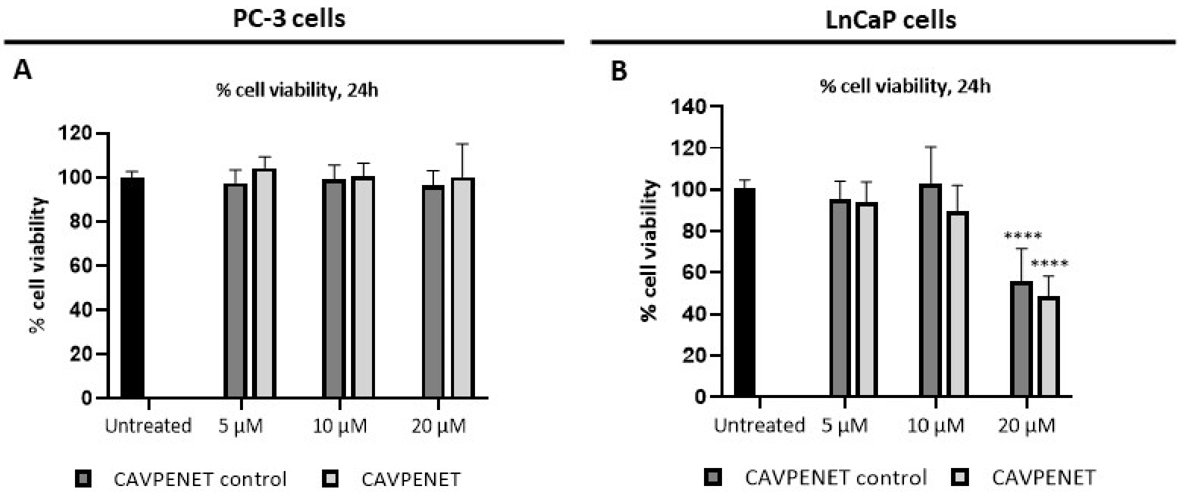

Supplementary Figure S1. Effect of CAVPENET control and CAVPENET bioportides on PC-3 (**A**) and LnCaP (**B**) cells viability. The cells were incubated with bioportides (5, 10 and 20µM) for 24h and cells' viability were evaluated using PrestoBlue cell viability assay. The percentage of cells viability were calculated through the ratio between treated and untreated conditions, considering untreated condition as 100% viability. The results are expressed as mean±SD from two independent experiments with five replicates/condition. **** $p < 0.0001$.

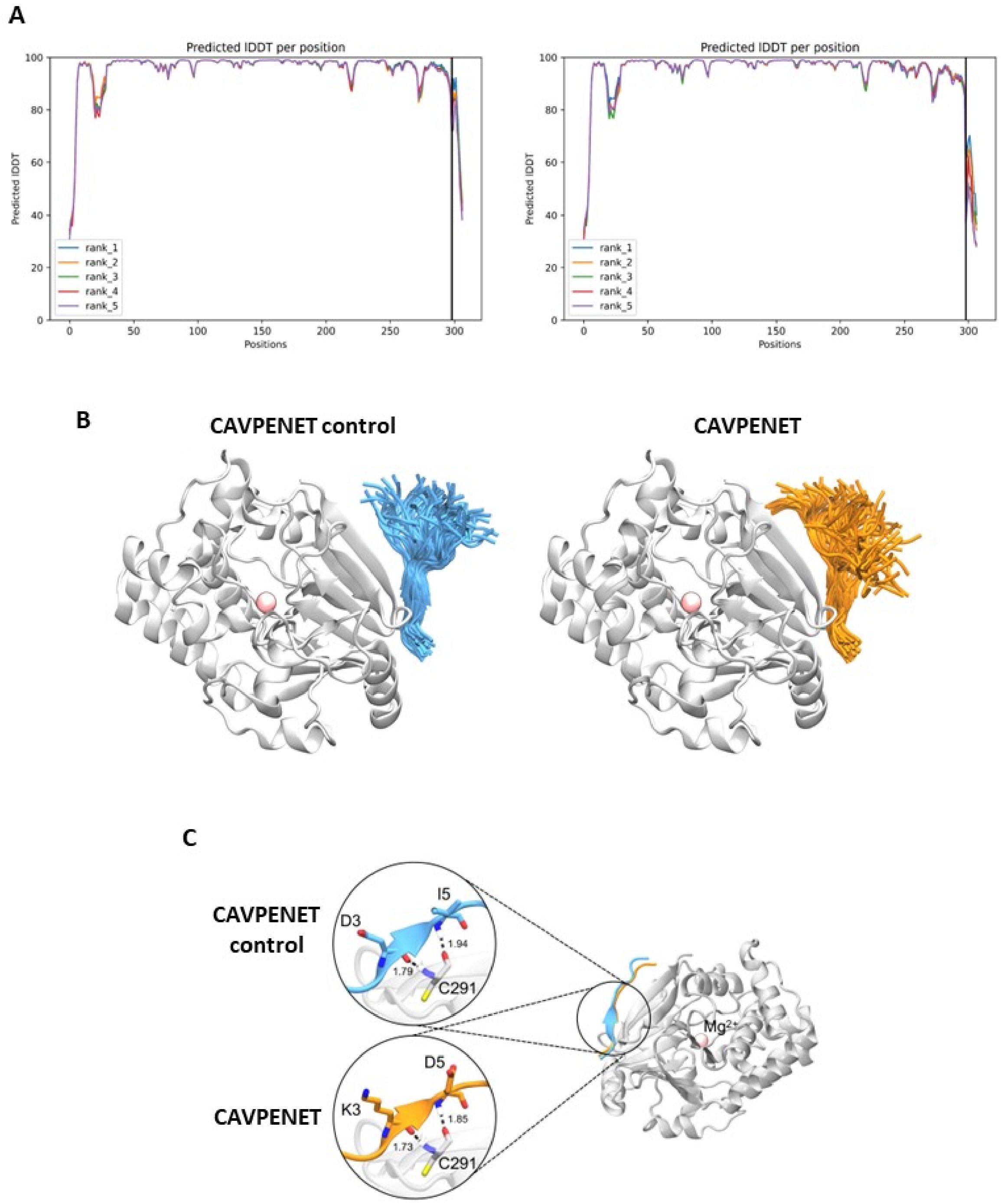

Supplementary Figure S2. (A) Predicted lDDT values per residue of the complex PP1/CAVPENET control (left) and PP1/CAVPENET (right) for the best 5 models were obtained by AlphaFold. The vertical black line (position 300) determines the end of PP1 and beginning of peptides. **(B)** Dynamic behaviour of CAVPENET control (cyan) and CAVPENET (orange) peptides, showing representative frames obtained from MD simulations. **(C)** Identification of similar hydrogen bonds (dotted black lines) formed by C291 backbone atoms from PP1 and residues in positions 3 and 5 of bioportides peptides. PP1, CAVPENET control and CAVPENET are shown as cartoons in gray, cyan and orange, respectively. Mg2+ ion is shown as a pink sphere, and residues participating in hydrogen bonds of backbone atoms are shown as sticks. Nitrogen, oxygen, and sulfur atoms are shown in blue, red, and yellow, respectively.

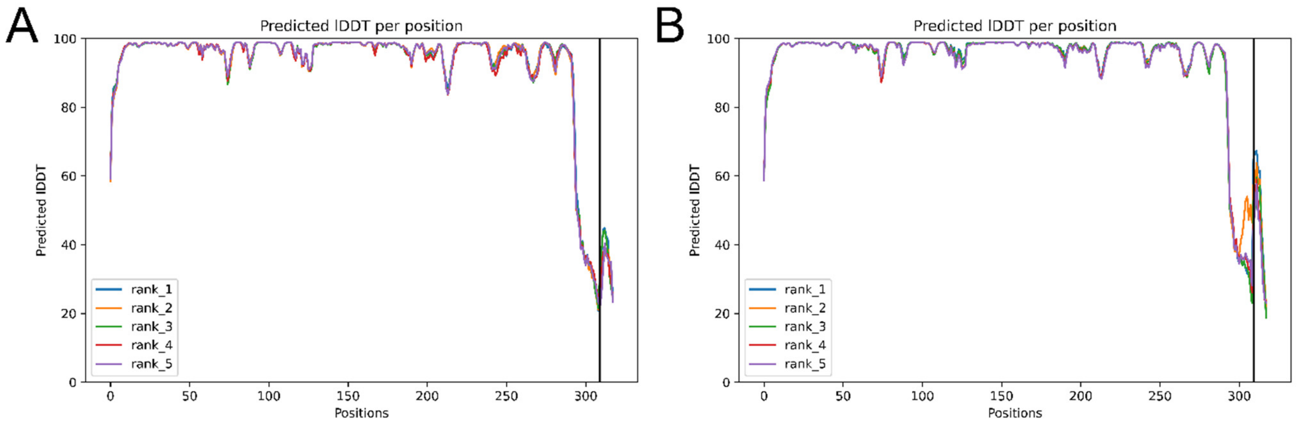

Supplementary Figure S3. Predicted lDDT values per residue of the complex PP2A/CAVPENET control **(A)** and PP2A/CAVPENET **(B)** for the best 5 models were obtained by AlphaFold. The vertical black line (position 300) determines the end of PP2A and beginning of peptides.

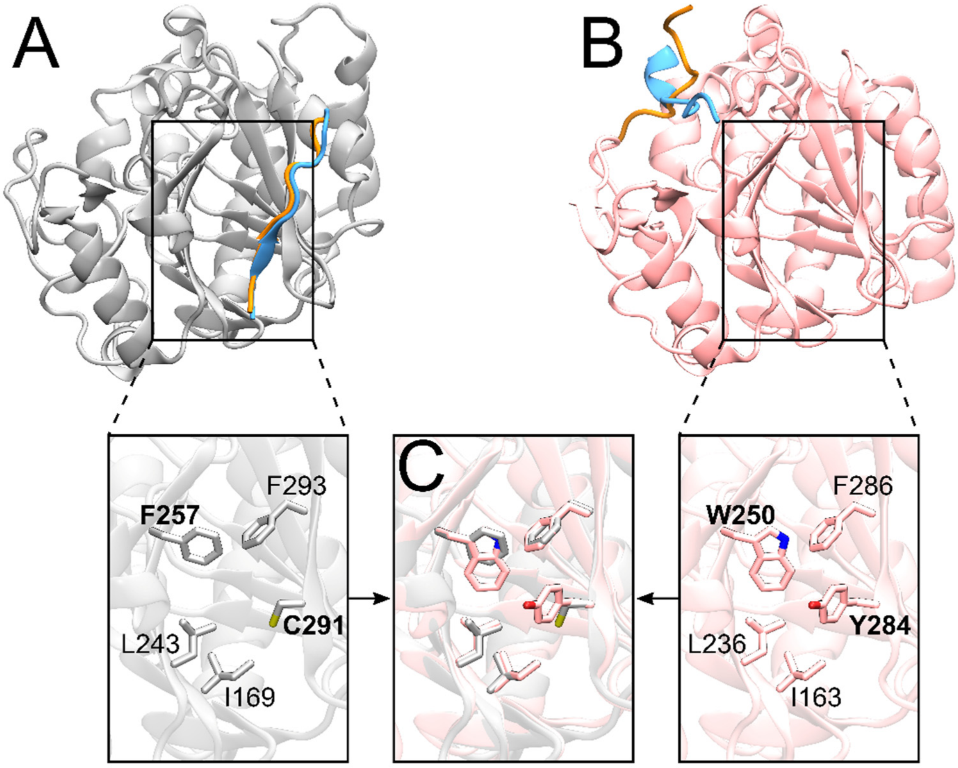

Supplementary Figure S4. Structural insights of the interaction of PP1 and PP2A and CAVPENET bioportides. The predicted structure of PP1 (gray) shows CAVPENET control (cyan) and CAVPENET (orange) interacting in a hydrophobic region, composed of I169, L243, **F257**, **C291** and F293 residues (zoomed panels) **(A)**. The same prediction was performed for PP2A (pink) interacting with the same peptides. The residues involved in the interaction were I163, L236, **W250**, **Y284**, and F286 **(B,C)**.
